# Emerging Variants of the Integrative and Conjugant Element ICE*Mh1* in Livestock Pathogens: Structural Insights, Potential Host Range, and Implications for Bacterial Fitness and Antimicrobial Therapy

**DOI:** 10.3389/fmicb.2019.02608

**Published:** 2019-11-12

**Authors:** Andrew Cameron, Rahat Zaheer, Tim A. McAllister

**Affiliations:** ^1^Faculty of Veterinary Medicine, University of Calgary, Calgary, AB, Canada; ^2^Lethbridge Research and Development Centre, Lethbridge, AB, Canada

**Keywords:** bovine respiratory disease, *Pasteurella multocida*, *Mannheimia haemolytica*, antimicrobial resistance, synergy, integrative and conjugative element, conjugative transposon, cattle

## Abstract

Horizontal gene transfer of integrative and conjugative elements (ICE) in bacterial pathogens of the bovine respiratory disease (BRD) complex has emerged as a significant cause of antimicrobial resistance (AMR) and therapeutic failure and mortalities in cattle. The aim of this study was to assess an AMR ICE occurring in *Pasteurella multocida* from a case of BRD, designated ICE*Mh1*^PM22^ for its structure and host genome insertion site, and to identify consequences for host fitness and antimicrobial therapy. The modular structure of ICE*Mh1*-like elements found in several related livestock pathogens was compared to ICE*Mh1*^PM22^, and the repertoire of cargo genes in variable ICE modules was functionally categorized. AMR genes were identified as frequent additions to the variable modules of ICE*Mh1*-like elements. Random PCR-based mapping of ICE*Mh1*^PM22^-genome junctions in transconjugants provided evidence that ICE*Mh1*^PM22^ integrates into the tRNA-leu for the UUG codon, and not into tRNA-leu for other codons. This was separately confirmed in the genomes of ICE*Mh1*-like-harboring livestock pathogens. Bacterial genera harboring receptive tRNA-leu^UUG^ were identified to establish the potential host range of ICE*Mh1*-like elements. ICE*Mh1*^PM22^-carrying transconjugants in *P. multocida* and *Mannheimia haemolytica* were less fit than isogenic strains without the ICE when grown without antimicrobial selection. This fitness cost was abrogated in the presence of subinhibitory concentrations of antimicrobials. Despite this cost, ICE*Mh1*^PM22^ was retained in transconjugants in extended culture. To identify possible therapeutic efficiencies, antimicrobial combinations were screened for synergistic interactions against AMR ICE*Mh1*^PM22^-carrying transconjugants. No antimicrobial combination tested exhibited synergistic interactions against AMR *P. multocida* or *M. haemolytica* harboring ICE*Mh1*^PM22^. In conclusion, this study provided information on the structural variation of ICE*Mh1*-like elements, refined the ICE insertion site and potential host range, and demonstrated the risk and consequences for AMR following horizontal transfer of ICE into BRD pathogens.

## Introduction

Integrative and conjugative elements (ICEs) are mobile genetic elements that can transfer autonomously by conjugation in bacteria. In general, ICEs can excise from the host chromosome to form a circular extrachromosomal intermediate which can be transferred and integrated into a receptive host (Johnson and Grossman, 2015). These widespread elements have increasingly been detected in bovine pathogens comprising the bacterial component of the bovine respiratory disease (BRD) complex (Michael et al., 2011b; Eidam et al., 2014). BRD bacteria are typically opportunistic pathogens which harmlessly reside in the nasopharynx, but upon exposure of cattle to stressors (e.g., weaning, transportation, feed changes) these bacteria can invade the respiratory tract and cause infection in conjunction with a variety of viral agents (Mosier, 2015). Serious BRD infections often lead to bovine pneumonia mortalities in calves. As a cause of significant economic loss to the cattle industry, BRD is typically prevented or treated with antimicrobials, including the use of in-feed antimicrobial prophylaxis (Cameron and McAllister, 2016). ICEs tend to have a modular structure, comprising modules for recombination and conjugation (Johnson and Grossman, 2015), but ICEs may also carry ‘cargo’ gene modules that are often advantageous to the recipient cell in terms of altering virulence, metabolic, and antimicrobial resistance capabilities. The first comprehensive reports of ICEs in BRD Pasteurellaceae described ICE*Pmu1* and ICE*Mh1*, in *Pasteurella multocida* strain 36950 (Michael et al., 2011a, b) and *Mannheimia haemolytica* strain 42548, respectively (Eidam et al., 2014). Recently, related ICE sequences have been described in other BRD Pasteurellaceae isolates (Klima et al., 2016; Beker et al., 2018). Of particular concern, these ICEs can harbor multiple resistance genes, including determinants capable of conferring resistance to most veterinary antimicrobials that are approved for the treatment of BRD (FDA, 2016). In addition to those Pasteurellaceae, ICE ICE*Pmu1*- and ICE*Mh1*-like sequences have also been detected in mortality-associated *Histophilus somni* (Bhatt et al., 2018), as well as in disease-associated *Pasteurellaceae* from swine suffering from respiratory infection (M. J. Hauglund et al., USDA, unpublished data). Furthermore, resistance genes found in these ICE are often highly related to AMR genes from other human and veterinary bacteria (Michael et al., 2011a, b; Eidam et al., 2014; Cameron et al., 2018).

With the increasing availability of BRD-associated bacterial genome sequences, it is now evident that related ICEs are widespread in the Pasteurellaceae, which differ primarily in their ‘cargo’(Beker et al., 2018). ICE*Pmu1*, the first BRD ICE characterized in *P. multocida*, was shown to integrate into a copy of the leucine tRNA (tRNA-leu) (Michael et al., 2011b). The chromosome-integrated form of ICE*Pmu1* was flanked by 13 bp direct repeats (5′-GATTTTGAATCAA-3′), which corresponded to the *attB* attachment site for site-specific recombination. In the circular form of ICE*Pmu1*, the direct repeat sequence is flanked by left and right ‘attachment’ sites, *attL* and *attR*, which are thought to be the recombination sites for a XerCD-like pair of tyrosine recombinases (Castillo et al., 2017). In the integrated form, the direct repeat and either *attL* or *attR* comprise the left and right terminals of the ICE. ICE*Mh1* also targets tRNA-leu, but is flanked by a shorter, but otherwise identical 11 bp direct repeat (5′-GATTTTGAATC-3′) (Eidam et al., 2014). Thus, ICE*Pmu1* and ICE*Mh1* seem to have evolved from a common ancestor, and a number of genes (particularly those for recombination and conjugation) are syntenous (Eidam et al., 2014). Both ICEs also harbor two separate cargo regions named ‘resistance region 1′ and ‘resistance region 2′ because these regions characteristically harbor genes for antimicrobial resistance. ICE*Pmu1* has 12 resistance genes: *strA* and *strB* (conferring resistance to streptomycin), *aphA1* (gentamicin), *sul2* (sulfonamides), *tet*(H) (tetracyclines), *floR* (phenicols), *erm*(42) (macrolides and lincosamides), *aadB* (gentamicin), *aadA15* (streptomycin and spectinomycin), *bla*_OXA–__2_ (β-lactams), and *msr*(E) and *mph*(E) (macrolides) (Michael et al., 2011a, b). ICE*Mh1* has only 5 resistance genes: *strA*, *strB*, *aphA1*, *sul2*, *tet*(H) (Eidam et al., 2014). Both ICEs are fully mobilizable and proven to integrate into *P. multocida*, *M. haemolytica*, and *E. coli* recipients (Klima et al., 2014).

The observation that ICE*Pmu1* and ICE*Mh1* can easily move between genera and species highlights the extreme risk for rapid acquisition of AMR in BRD bacteria via horizontal gene transfer. The acquisition of these AMR genes poses an obvious threat to effective antimicrobial therapy, and could exacerbate economic losses associated with cattle mortalities. Greater knowledge of basic ICE biology is necessary to understand their potential host range, including acquisition by human pathogens, and the threat they pose to the efficacy of antimicrobial therapy. We explored these attributes using isogenic transconjugants of an ICE*Mh1*-like element (ICE*Mh1*^PM22^) previously identified in a bovine isolate (*P. multocida* PM22) from a BRD mortality (Klima et al., 2014). The objectives of this study were to (I) refine the insertion site of ICE*Mh1*-like elements, (II) predict host range based on the insertion site, (III) explore the effect of ICE*Mh1*^PM22^ acquisition on host fitness, and (IV) identify any synergistic antimicrobial combinations effective against bacteria harboring ICE*Mh1*^PM22^. We tested for synergy using antimicrobials against which PM22 was both non-susceptible and susceptible to identify any combination capable of restoring susceptibility. We hypothesized that this information could be used to ensure prudent antimicrobial usage, inform future BRD mitigation strategies, and identify the potential risk in other areas of human and veterinary medicine.

## Materials and Methods

### Bacterial Strains and Growth Conditions

*P. multocida* PM22 was initially isolated following lung tissue necropsy of a beef cattle BRD mortality (acute fibrinous pneumonia) in a Texas feedlot (Klima et al., 2014). Pasteurellaceae were routinely grown on tryptic soy agar (TSA) blood agar (Dalynn Biologicals, Calgary, AB) or Brain Heart Infusion (BHI) agar (BD Difco, Mississauga, ON). *E. coli* were maintained with Luria-Bertani (LB) or Mueller-Hinton II (MH; cation-adjusted) agar/broth (BD Difco) supplemented with antibiotics where appropriate. All susceptibility testing was performed in MH broth. Spontaneous rifampin-resistant (Rif^R^) mutants of *P. multocida* CCUG 17976 and *M. haemolytica* ATCC 33396 were obtained by plating OD_600_ 2.0 on BHI supplemented with 50 mg/L rifampin (rifampicin; MilliporeSigma, Oakville, ON, United States), followed by incubation for ∼48 h at 37°C. To generate luciferase-expressing *E. coli* DH5α, the pAKlux2 plasmid (Karsi and Lawrence, 2007) was electroporated into *E. coli* DH5α and transformants were selected on and routinely maintained on LB agar supplemented with 100 mg/L of ampicillin (MilliporeSigma).

### Conjugation and Mapping of ICE Insertion Junctions by Random PCR

Bacterial conjugation assays were performed as previously described (Klima et al., 2014), with minor modifications. Briefly, in three independent experiments, OD_600_ 1.0 equivalents of *P. multocida* PM22 and Rif^R^ *P. multocida* CCUG 17976 or *M. haemolytica* ATCC 33396 were mated in 100 μL of BHI broth spotted on TSA blood agar, incubated aerobically at 37°C for 4 h, harvested and then plated on BHI agar supplemented with rifampin (50 mg/L), tetracycline (10 mg/L; MilliporeSigma), and spectinomycin (100 mg/L; MilliporeSigma). Six colonies were selected from each mating (18 in total for each species) and verified for ICE*Mh1*^PM22^ acquisition by susceptibility testing and PCR screening (Klima et al., 2014). To further ensure that *P. multocida* transconjugants were not spontaneous, Rif^R^ mutants of *P. multocida* PM22, transconjugants were also tested for ceftiofur MIC (the cephalosporin resistance phenotype is not transferred to transconjugants). To map the ICE*Mh1*^PM22^ insertion site, transconjugant DNA was purified (DNeasy Blood and Tissue kit, Qiagen, Montreal, QC) and amplified using the CEKG set of nested and random (i.e., containing degenerate sequence) oligonucleotides in combination with nested ICE*Mh1*^PM22^-specific oligonucleotides: PM22-A1: 5′-CACCTTTAGTTGAAGCAATAG-3′; PM22-A2, 5′-TGGTAA AAGGTTTGTTTGTAC-3′; PM22-B1, 5′-ATCGTAGTAAGTG TGTATTTG-3′; and PM22-B2 5′-ATTATTTGAACAGTTC TACGC-3′ (Eurofins Genomics, Toronto, ON) in tandem rounds of PCR as described by Salama et al. (2004). Resulting amplicons were gel-purified (Zymoclean Gel DNA Recovery Kit, Zymo Research, Irvine, CA, United States) and sequenced (Eurofins Genomics).

### Growth Curves and Co-culture Competitions

Growth curves of Rif^R^ *P. multocida* CCUG 17976 and *M. haemolytica* ATCC 33396 and their isogenic ICE*Mh1*^PM22^ transconjugants were performed in 200 μL volumes of MH broth inoculated at an OD_600_ of 0.005 in 96-well plates (Nunc) sealed with gas-permeable film (Breathe-Easy sealing membrane, MilliporeSigma). Bacterial growth, with shaking at 37°C, was monitored continuously for 12 h in a plate-reader (Biotek HT Synergy). For 2-strain co-culture experiments, luciferase-expressing *E. coli* DH5α pAKlux2 and test strains (including DH5α without pAKlux2 as a control) were each inoculated at an OD_600_ of 0.0025 (i.e., total OD_600_ of ∼0.005) into black clear bottom 96-well plates (Nunc, Thermo-Fisher Scientific, Ottawa, ON, Canada). Bacterial growth was monitored as above, with the addition of luminometry. The maximum light produced (in relative light units, RLU) in each competition was recorded, and used to generate a competitive index (i.e., RLU_Teststrains vs. DH5α pAKlux2_/RLU_DH5α vs. DH5α pAKlux2_). Thus, values <1 indicated that test *E. coli* DH5α pAKlux2 was outperformed compared to an *E. coli*-only control. For long-term repeated passage experiments to assess if ICE*Mh1*^PM22^ could be lost from the host strain, ICE*Mh1*^PM22^ transconjugants were inoculated into 2 mL of MH broth or MH broth supplemented with 0.5 MIC (subinhibitory for the susceptible WT) oxytetracycline (0.125 mg/L), spectinomycin (32 mg/L), or tylosin (16 mg/L) in a sterile 96-well block (Greiner Bio-One, Monroe, NC), and sub-cultured (1/100) every ∼3 d into fresh media supplemented with the same antimicrobials for 150 d. To assess for loss of ICE*Mh1*^PM22^, CFU from OD_600_ 0.1-equivalents from each culture were enumerated in parallel on MH agar (total count) and MH agar supplemented with each antimicrobial at concentrations selective for ICE*Mh1*^PM22^: oxytetracycline (10 mg/L); spectinomycin (512 mg/L); or tylosin (64 mg/L). Likewise, the effect of subinhibitory concentrations on co-culture competitions with luciferase-expressing *E. coli* was assessed with 0.5 MICs (for WT strains) of oxytetracycline (0.125 mg/L) and spectinomycin (32 mg/L) as above for co-cultures. Tylosin was not tested because *E. coli* is intrinsically resistant to macrolides.

### MIC Determination and Checkerboard Assay Antimicrobial Synergy Screening

Minimum inhibitory concentrations (MICs) were determined for the Rif^R^ and isogenic ICE*Mh1*^PM22^ *P. multocida* CCUG 17976 and *M. haemolytica* ATCC 33396 transconjugants according to the CLSI approved standard M07 - Methods for dilution antimicrobial susceptibility tests for bacteria that grow aerobically (CLSI, 2018). *Escherichia coli* ATCC 25922 and *E. faecalis* ATCC 29212 were used as quality control organisms for susceptibility testing. Susceptibility designations for *P. multocida* PM22 were previously assigned (Klima et al., 2014) in accordance with the CLSI approved standard M31-A3 – Performance standards for antimicrobial disk and dilution susceptibility tests for bacteria collected from animals (CLSI, 2008). Antimicrobials tested were purchased from MilliporeSigma (oxytetracycline hydrochloride, chlortetracycline, tilmicosin, gamithromycin, spectinomycin dihydrochloride pentahydrate, penicillin G sodium salt, ampicillin, ceftiofur hydrochloride, sulfamethoxazole, enrofloxacin, tiamulin fumarate, clindamycin hydrochloride and florfenicol) or from AlfaAesar (Haverhill, MA; tylosin tartrate, neomycin sulfate hydrate and sulfamethazine). Briefly, antimicrobial stocks were prepared immediately prior to testing with respect to potency in the appropriate solvent and filter sterilized where appropriate. Broth microdilution assays were performed in triplicate in 96-well plates in a total volume of 100 μL of MH broth containing 2-fold dilution series of each antimicrobial and inoculated with 5 μL of each strain tested (total inoculum of ∼5 × 10^4^ CFU). Plates were grown aerobically at 37°C for 20 h and MIC was registered as the lowest concentration inhibiting visually detectable growth. For checkerboard synergy assays, two-dimensional arrays of serial concentrations (8 × 8 wells; 2-fold dilution series) of antimicrobials were created for two experiments: (I) all combinations of 5 antimicrobials (i.e., oxytetracycline, spectinomycin, tilmicosin, tylosin, and sulfamethazine) representing major antimicrobial classes to which transconjugants were non-susceptible, and (II) selected combinations of ‘non-susceptible’ antimicrobials (i.e., oxytetracycline, spectinomycin, and tylosin) with antimicrobials to which ICE*Mh1*^PM22^ transconjugants were susceptible (i.e., penicillin G, enrofloxacin, florfenicol, ceftiofur, and chlortetracycline). Growth and sterility controls were included in each checkerboard assay, which were performed in triplicate. MICs were interpreted by eye and OD_600_ was measured in a plate reader to facilitate calculation of a fractional inhibitory concentration index (FICI). For discrepancies between replicates, the highest antimicrobial concentration determined the MIC. The FIC for each antimicrobial (A or B) was determined by the MIC of the antimicrobial in combination and divided by the MIC of the drug alone, according to:

$$F\,I\,C\,I=F\,I\,C_{A}+F\,I\,C_{B}=\left(\frac{M\,I\,C_{A+B}}{M\,I\,C_{A}}\right)+\left(\frac{M\,I\,C_{B+A}}{M\,I\,C_{B}}\right)$$

Interactions were conservatively interpreted as ‘synergistic’ (FICI ≤ 0.5) or ‘no interaction’ (FICI > 0.5–4.0) in line with synergy testing guidelines in the *Journal of Antimicrobial Chemotherapy* (Odds, 2003).

### Bioinformatics and Statistical Analyses

Statistical tests and graphing were performed with Sigmaplot 13.0 (Systat Software Inc.), with error bars indicating standard error of the mean (SEM). ICE*Mh1*-like-containing Pasteurellaceae genomes were identified by default BLAST alignment to ICE*Mh1*^PM22^, using only results with >80% sequence identity and >50% query coverage and eliminating isogenic or partial sequences. Sequence manipulations and alignments were performed in Geneious 8.1.9 using default MUSCLE parameters or progressive Mauve alignment (Darling et al., 2010) for identification of conserved and variable regions (and genes) in ICE*Mh1*-like sequences. Gene Annotations in 41 ICE*Mh1*-like sequences were standardized by re-annotation with PROKKA (Seemann, 2014) and manually scrutinized for putative function. Trees were constructed with PhyML (Jukes-Cantor substitution model). Sequence logos were produced with WebLogo (Crooks et al., 2004). Sequence similarity between ICE*Mh1*-like sequences was visualized with Circos (Krzywinski et al., 2009). Predicted tRNA structure and identification was performed with tRNAscan-SE (Chan and Lowe, 2019). To identify bacteria with tRNA-leu^UUG^ containing the conserved direct repeat and palindrome sequences, a BLAST search was performed using the *trnL2* (tRNA-leuUUG) sequence from *M. haemolytica* M42458 with parameters: Program, blastn; Word size, 11; Expect value, 10; Hitlist size, 20,000; Match/Mismatch scores, 2/-3; Gapcosts, 5/2. This resulted in 9604 hits/alignments, which were filtered for those containing the ICE*Mh1*-associated direct repeat (i.e., *attB*) and palindrome (with 100% identity). From this, a taxonomic report was generated to show bacterial genera.

## Results

### General Structure of the ICE*Mh1*-like Variant ICE*Mh1*^PM22^ From a BRD-Associated *P. multocida* Isolate

**FIGURE 1:**
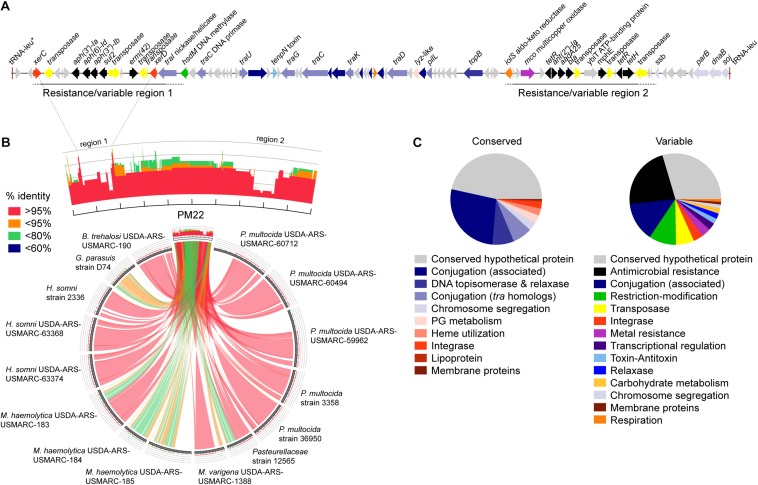
Genomic characterization of the ICE*Mh1*-like variant ICE*Mh1*^PM22^. **(A)** Gene schematic of ICE*Mh1*^PM22^ with genes shown as arrows and colored by functional characteristics, as shown in panel **(C)**. **(B)** Comparison of ICE*Mh1*^PM22^ with selected Pasteurellaceae genomic ICE sequences. In some cases, the ICE region cannot be delimited by the identification of the flanking direct repeat and includes sequences likely external to the ICE. Histograms represent the frequency and sequence identity (color) of alignments to ICE*Mh1*^PM22^: red, >95% sequence identity; orange, >95%; green, <80%; blue, <60%. **(C)** Frequencies of annotated genes by functional characteristics found in conserved (left) and variable (right) regions in 41 ICE*Mh1*-like-containing Pasteurellaceae (with >80% sequence identity and >50% query coverage alignment to ICE*Mh1*^PM22^).

ICE*Mh1*^PM22^ is a 77,786 bp element in *P. multocida* PM22 (accession # CP045724) bound by two copies of a tRNA for leucine (tRNA-leu), one of which is disrupted and potentially non-functional (on left, Figure 1A) whereas the second copy is full-length and likely functional (on right, Figure 1A). There is also a third disrupted copy of tRNA-leu upstream of the start codon of the full-length functional tRNA-leu (not shown). The ICE*Mh1*^PM22^ sequence may encode for ∼94 proteins that have been annotated with putative functions or as conserved hypothetical proteins. Similar to the ICE*Mh1* prototype in *M. haemolytica* strain 42548 (Eidam et al., 2014) and ICE*Pmu1* in *P. multocida* strain 36950 (Michael et al., 2011b), and other Pasteurellaceae ICE (Beker et al., 2018), ICE*Mh1*^PM22^ harbors multiple antimicrobial resistance genes/gene cassettes interspersed with numerous putative transposases in two discrete ‘resistance regions’. The first (left) region—resistance region 1, encodes aminoglycoside (*aph(3′)-Ia*, *aph(6)-Id*, *aph(3″)-Ib*), sulfonamide (*sul2*), and macrolide (*erm42*) resistance genes, and the second—resistance region 2—encodes genes for tetracycline (*tetH*, *tetR*), aminoglycoside (*ant(2″)-Ia*, *aadA25*), macrolide (*mphE*) resistance, and a potential extended-spectrum β-lactamase (*bla*_OXA–__2_) previously shown to be phenotypically non-functional in transconjugants (Klima et al., 2014). This two-region structure appears to be widely present in other ICE*Mh1*-like elements found in 41 Pasteurellaceae genome sequences, encompassing the livestock pathogens *P. multocida*, *M. haemolytica*, *Histophilus somni*, *Bibersteinia trehalosi*, and *Glaesserella parasuis* (Figure 1B). Also routinely present in ICE*Mh1*-like sequences are putative tyrosine recombinases (integrases), sometimes annotated as XerC and XerD, members of which are thought to work in tandem by mediating site-specific recombination (i.e., insertion and excision of the ICE) using two recognition sites which separately constitute the *attL* and *attR* sites in integrated ICE, and which are found on either side of a central attachment site involved in crossover (Michael et al., 2011b; Johnson and Grossman, 2015; Castillo et al., 2017). Alignment of *xerC* and *xerD* sequences found in 41 ICE*Mh1*-like elements indicates a high degree of similarity (alignment identity: *xerC*, 77.9% identity; *xerD* 82.4% identity). XerC and XerD are likely part of the core or conserved backbone of ICE*Mh1*-like elements, as are genes annotated as part of the conjugation machinery, such as the *tra* genes (Johnson and Grossman, 2015). Most of these were usually present in the central region of ICE*Mh1*-like elements with >95% identity. We further examined the gene content in conserved and variable regions of 41 ICE*Mh1*-like elements by extracting gene annotations from MAUVE-aligned sequences, defining ‘conserved’ as alignments found in >50% of ICE*Mh1*-like elements with >80% sequence identity. ‘Conserved’ alignments harbored the majority of the conjugation machinery, such as *tra* and conjugation-associated proteins of unknown function, and DNA topoisomerases, integrases, and chromosome segregation factors potentially involved in ICE mobilization (Figure 1C). ‘Variable’ alignments contained more functionally diverse genes, including genes for antimicrobial and metal resistance, transposition, transcriptional regulation, potentially expanded metabolic functions (e.g., carbohydrate utilization and other respiratory genes), and toxin-antitoxin systems.

### ICE*Mh1*-like Elements Insert Into the tRNA-leu for the UUG Codon

**FIGURE 2:**
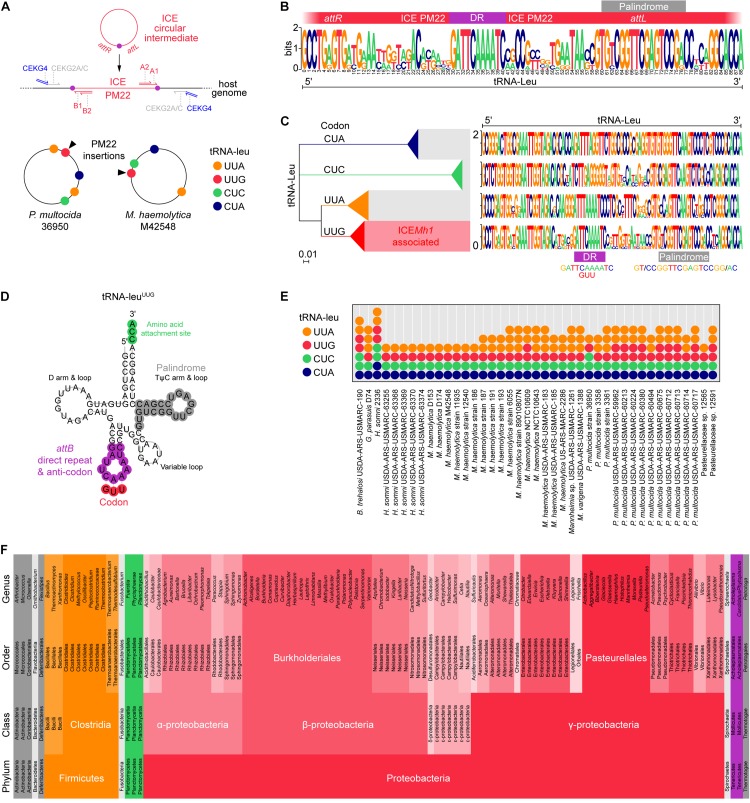
Identification of tRNA-leu^UUG^ as the putative *attB* insertion site of ICE*Mh1*-like elements and predicted host range. **(A)** Schematic for ICE orientation and random PCR mapping of ICE*Mh1*^PM22^ junctions in 18 *Mannheimia haemolytica* and 18 *Pasteurella multocida* transconjugants. ICE*Mh1*^PM22^ insertions in every transconjugant were identical and aligned to a specific tRNA-leu in the genomes of *P. multocida* strain 36950 and *M. haemolytica* M42548. **(B)** Sequence logo of left and right MUSCLE-aligned junctions identified in 41 Pasteurellaceae harboring ICE*Mh1*-like sequences. The left and right ends of ICE*Mh1*-like variants contain a conserved direct repeat (DR; 5′-GATTCAAAATC-3′) *attL* conserves the tRNA TψC loop’s imperfect palindrome (5′-CGGTTCGAGTCCG-3′). The *attL* and *attR* sites are designated with respect to ICE*Pmu1* in Michael et al. (2011b). **(C)** Neighbor-joining tree of all tRNA-leu from 41 Pasteurellaceae harboring ICE*Mh1*-like sequences. All ICE*Mh1*-like insertions were associated with tRNA-leu^UUG^. **(D)** Predicted structure of tRNA-leu^UUG^ showing presumptive ICE*Mh1 attB* attachment site (also encoding the tRNA anticodon) and palindrome. **(E)** Frequencies of tRNA-leu codon types in 41 Pasteurellaceae harboring ICE*Mh1*-like sequences. **(F)** Predicted host range of ICE*Mh1*-like elements based on tRNA-leu^UUG^ alignment by BLAST and strict (100% identity) conservation of direct repeat and palindrome. Colors are arbitrary.

It was previously shown that ICE*Mh1*-like elements insert into tRNA-leu genes in *P. multocida* and *M. haemolytica* (Michael et al., 2011b; Eidam et al., 2014). The terminal ends of ICE*Mh1* (and the cognate *attB* genome attachment site) were identified as a direct repeat (5′-GATTCAAAATC-3′) (Eidam et al., 2014). Given that there are multiple copies of tRNA-leu in most bacterial genomes, we initially thought that any could be receptive to ICE*Mh1*-like elements. To determine this, we mapped ICE*Mh1*^PM22^ junctions in transconjugants using degenerate (random) PCR oligonucleotides (Salama et al., 2004) with oligonucleotides specific for the terminal ends of ICE*Mh1*^PM22^ (Figure 2A). Following sequencing and alignment of PCR-amplified junctions, ICE*Mh1*^PM22^ transconjugants in *P. multocida* CCUG 17976 and *M. haemolytica* ATCC 33396 were found to have identical insertion sites, which we identified as a single tRNA-leu in each strain as shown mapped to the known genome sequences of *P. multocida* 36950 and *M. haemolytica* M42548 (Figure 2A). Furthermore, alignment of the left and right junctions in 41 ICE*Mh1*-like-containing genomes indicated that those ICEs had exclusively inserted into tRNA-leu sequences in which both the direct repeat and an imperfect palindrome (5′-CGGTTCGAGTCCG-3′) were present (Figure 2B). To characterize this further, we examined all the tRNA-leu present in the 41 ICE*Mh1*-like-containing genomes and found that these sequences could be categorized by multiple alignment phylogeny into four types in both *P. multocida* and *M. haemolytica*; representing 4 of the 6 possible anticodons for leucine (Figure 2C). This was consistent with data from the GtRNAdb tRNA databases (Chan and Lowe, 2015) which indicates that these bacteria typically encode at least 4 tRNAs for the leucine codons CUA, CUC, UUA, and UUG. The other two tRNA-leu codons, CUG and CUU, are absent in currently known *P. multocida and M. haemolytica* genomes. Only tRNA-leu^UUG^ was integrated with ICE*Mh1*-like elements in the 41 genomes studied. The predicted structure of tRNA-leu^UUG^ suggests that the ICE direct repeat constitutes the tRNA anticodon loop, and the palindrome is the basis of the T-loop (or TψC loop) ribosome recognition site (Figure 2D). Our analysis of ICE*Mh1*^PM22^ junctions also suggested that ICE insertion leaves one functional and one partial (disrupted) copy of tRNA-leu^UUG^ (not shown). In the 41 ICE*Mh1*-like-containing genomes, at least 1 functional copy of each tRNA was present, although sometimes full-length tRNA-leu^UUG^ was present twice (Figure 1E). In all cases where two functional copies of tRNA-leu^UUG^ were present, the copies were located directly on either side of the ICE insertion. We also observed that one terminus (i.e., direct repeat) of the ICE was absent in some ICE*Mh1*-like-containing genomes, potentially indicating ICEs incapable of mobilization (not shown).

### Predicted Host Range of ICE*Mh1-*like Elements

Given the conservation of the tRNA-leu^UUG^ direct repeat and palindrome in the terminal ends of ICE*Mh1*-like elements, we used these as criteria for *in silico* prediction of potential host range. The full-length *M. haemolytica* tRNA-leu^UUG^ sequence was used as the basis for a *blastn* nr database search optimized for short input sequences. Following retrieval of 9,604 BLAST alignments, tRNA-leu containing the exact direct repeat and palindrome sequence were filtered, and a taxonomic report showing bacterial genera with members with those attributes was constructed (Figure 2F). As expected, the phylum with the greatest number of genera harboring similar tRNA-leu were the Proteobacteria, specifically γ-Proteobacteria, of which Pasteurellales were most abundant (an Order which includes *P. multocida* and *M. haemolytica*, and some human pathogens, including *Haemophilus* spp.). Enterobacterales were also frequently represented, and included many well-known food-borne and cattle-associated pathogens such as *Escherichia coli* and *Salmonella* spp. For *E. coli*, only some strains harbored the receptive tRNA-leu sequence, suggesting that only some members of any genus might be capable of transconjugation. Burkholderiales (β-proteobacteria) also harbored similar tRNA-leu, suggesting the potential for ICE*Mh1*-like horizontal gene transfer to *Burkholderia* spp. and *Bordetella* spp. Likewise, bacteria from α-, δ-, and ε-Proteobacteria were also represented and may be receptive to ICE*Mh1*, such that cattle-associated food-borne bacteria like *Campylobacter* could conjugate with ICE-bearing Pasteurellaceae. Some Gram-positive Firmicutes, specifically Clostridia, were also host to ICE-receptive tRNA-leu. Thus, if the tRNA-leu^UUG^ sequence was the only requirement for mobilization and integration, ICE*Mh1* might be capable of crossing into these Gram-positive organisms. At present, it has only been experimentally confirmed that ICE*Mh1*^PM22^ can transfer into Rif^R^ *P. multocida* and *M. haemolytica* (Klima et al., 2014), and *E. coli* strains K-12 and DH5α (not shown).

### ICE*Mh1*^PM22^ Acquisition Confers a Fitness Cost in Laboratory Conditions

**FIGURE 3:**
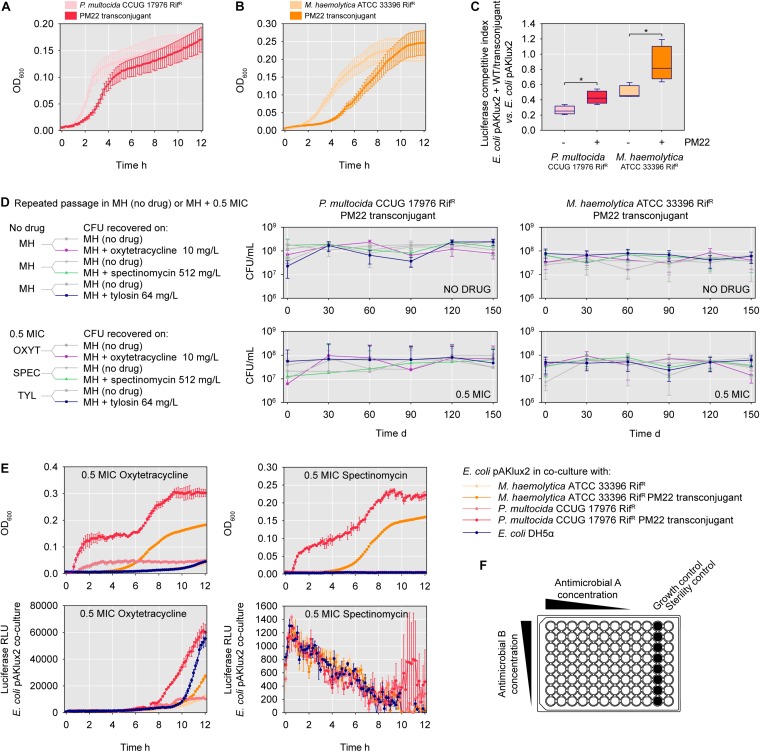
ICE*Mh1*^PM22^ fitness costs, addiction, and consequences for antimicrobial resistance in transconjugants. **(A)** Growth curves of *P. multocida* CCUG 17976 (spontaneous rifampin-resistant mutant) and isogenic ICE*Mh1*^PM22^ transconjugant. Mean of 4 biological replicates with SEM. **(B)** Growth curves of *M. haemolytica* ATCC 33396 (spontaneous rifampin-resistant mutant) and isogenic ICE*Mh1*^PM22^ transconjugant. Mean of 4 biological replicates with SEM. **(C)** Luciferase-based competition index from co-cultures of ICE*Mh1*^PM22^ transconjugants and *E. coli* DH5α harboring the luciferase expression plasmid pAKlux2. Mean of 4 biological replicates with SEM; t-test, ^∗^*P* ≤ 0.05. **(D)** Long-term repeated passage of ICE*Mh1*^PM22^ transconjugants. Transconjugants were grown in MH (no drug) or MH + 0.5 MIC (subinhibitory; MIC for susceptible *P. multocida* CCUG 17976 or *M. haemolytica* ATCC 33396 WT) for 150 days and plated on media with or without selective concentrations of indicated antimicrobials (i.e., 0.5 MIC for non-susceptible isogenic transconjugants). Mean of 3 biological replicates with SEM. **(E)** Upper panel: growth curves of co-cultures of *E. coli* DH5α pAKlux2 and *E. coli* DH5α, *P. multocida* CCUG 17976 ICE*Mh1*^PM22^, or *M. haemolytica* ATCC 33396 ICE*Mh1*^PM22^ in subinhibitory (0.5 MIC for susceptible *P. multocida* CCUG 17976 or *M. haemolytica* ATCC 33396) concentrations of oxytetracycline (left) or spectinomycin (right). Mean of 3 biological replicates with SEM. Lower panel: detection of *E. coli* luciferase production in co-cultures (as above) with either oxytetracycline (left) or spectinomycin (right). **(F)** Schematic of checkerboard synergy assay for drug interaction screening.

*M. haemolytica* ATCC 33396 transconjugants harboring ICE*Mh1*^PM22^ were noticeably slower-growing than the isogenic WT on agar media supplemented with selective concentrations of antimicrobials. To assess the effect of ICE acquisition on growth parameters, we performed OD_600_-based growth curves for Rif^R^ *P. multocida* CCUG 17976 and *M. haemolytica* ATCC 33396, and their respective ICE*Mh1*^PM22^ transconjugants. The transconjugant of Rif^R^ *P. multocida* CCUG 17976 exhibited both an extended lag phase and decreased growth rate compared to the ICE-minus strain (Figure 3A). Likewise, the transconjugant of the Rif^R^ *M. haemolytica* ATCC 33396 strain exhibited an extended lag phase, but did not have a significantly different growth rate (Figure 3B). To further explore the fitness costs of ICE*Mh1*^PM22^ integration, we competed the isogenic Rif^R^ WT and transconjugants against *E. coli* DH5α expressing luciferase, and monitored light production to assess for *E. coli* fitness against all four strains (Figure 3C). In all cases, the Pasteurellaceae inhibited *E. coli* light production. However, competitions with strains harboring ICE*Mh1*^PM22^ resulted in increased light production relative to the isogenic WT strains, also suggesting that ICE*Mh1*^PM22^ confers a fitness cost on the host.

### ICE*Mh1*^PM22^ Was Retained in Transconjugants Following Repeated Laboratory Passage

Given the apparent cost of ICE*Mh1*^PM22^ carriage, we hypothesized that hosts might lose the ICE following extended passage in non-selective conditions (i.e., without antimicrobials). To test this, the ICE*Mh1*^PM22^ transconjugants for Rif^R^ *P. multocida* CCUG 17976 and *M. haemolytica* ATCC 33396 were repeatedly passaged over 150 days in media without antimicrobials or supplemented with sub-inhibitory concentrations of oxytetracycline, spectinomycin, or tylosin. The chosen concentrations were sub-inhibitory (0.5 MIC) to the parental WT strains. Every 30 days, CFU from each repeated passage were enumerated on MH agar (total CFU) and MH agar supplemented with each antimicrobial at a selective concentration (i.e., at a concentration where non-susceptibility was conferred by the ICE*Mh1*^PM22^). The presence of ICE*Mh1*^PM22^ was also confirmed in selected isolated colonies by PCR (not shown). This showed that repeated passage of ICE*Mh1*^PM22^-carrying strains did not result in any measurable loss of the ICE (Figure 3D). In all cases there was no statistical difference between total and non-susceptible CFU counts in any repeated culture experiment. Thus, despite the fitness cost, ICE*Mh1*^PM22^ was retained in the absence of selection in these experiments.

### The Presence of ICE*Mh1*^PM22^ Altered Collective Resistance to Sub-Inhibitory Antimicrobial Concentrations

Given that previous work had shown that ICE*Mh1*^PM22^ could transfer into *E. coli*, we attempted to monitor transconjugation dynamics using luciferase-expressing *E. coli* in co-culture with Rif^R^ WT or ICE*Mh1*^PM22^ transconjugants under antimicrobial pressure. We thought that increased luciferase production might indicate conjugation events. Therefore, oxytetracycline (0.125 mg/L) and spectinomycin (32 mg/L) were selected as bacteriostatic antimicrobials and used at 0.5 MIC for both *P. multocida* and *M. haemolytica* Rif^R^ WT. At these concentrations, oxytetracycline was sub-inhibitory to *E. coli* DH5α pAKlux2 (MIC: ∼1 mg/L), but spectinomycin was inhibitory (MIC: ∼16 mg/L). Co-cultures of the transconjugants or Rif^R^ WT with *E. coli* DH5α pAKlux2 were monitored for OD_600_ and luciferase production over 12 h. For oxytetracycline, increased luciferase production was observed for both *P. multocida* and *M. haemolytica* ICE*Mh1*^PM22^ co-cultures compared to the ICE-minus Rif^R^ WT (Figure 3E, left). For spectinomycin, luciferase production was observed only in co-cultures of *E. coli* and the ICE*Mh1*^PM22^ *P. multocida* transconjugant (Figure 3E, right). However, increased luciferase production was not due to *E. coli* acquisition of ICE*Mh1*^PM22^, as non-susceptible *E. coli* could not be recovered on media supplemented with either tetracycline or spectinomycin at concentrations known to be selective for ICE*Mh1*^PM22^ *E. coli* transconjugants (not shown). These findings suggest that the presence of ICE*Mh1*^PM22^ negatively affected the efficacy of these antimicrobials against *E. coli* in co-culture, without horizontal gene transfer.

### Antimicrobials Used to Treat BRD Exhibit No Synergistic Interactions Against ICE*Mh1*^PM22^-Bearing *P. multocida* and *M. haemolytica*

Lastly, because ICE*Mh1*^PM22^ harbors an extensive repertoire of antimicrobial resistance genes for drugs in frequent and common use for BRD, we investigated whether or not combination antimicrobial therapies were effective against *P. multocida* or *M. haemolytica* ICE*Mh1*^PM22^ transconjugants. Checkerboard antimicrobial synergy assays (Figure 3F) were performed to test for (I) synergism between combinations of two antimicrobials against which ICE*Mh1*^PM22^ confers non-susceptibility (i.e., oxytetracycline, spectinomycin, tilmicosin, tylosin, and sulfamethazine; Table 1), and (II) synergism between selected combinations of antimicrobials against which ICE*Mh1*^PM22^ confers non-susceptibility (i.e., oxytetracycline, spectinomycin, and tylosin) and those that it does not (i.e., penicillin G, enrofloxacin, florfenicol, ceftiofur, and chlortetracycline). Following calculation and strict interpretation of the FICI scores for each combination, we found that no pairwise combination of oxytetracycline, spectinomycin, tilmicosin, tylosin, and sulfamethazine demonstrated synergism (Table 2). Likewise, no combination of a non-effective antimicrobial with any effective antimicrobial tested exhibited synergy (Table 3), and when present, susceptibility was due to the action of only the effective antimicrobial.

**TABLE 1:** MIC changes in transconjugants.

**Antimicrobial**	**MIC*^a^***			
	
	***M. haemolytica* ATCC 33396*^b^***	***M. haemolytica* ATCC 33396*^b^* × PM22 transconjugant*^c^***	***P. multocida* CCUG 17976*^b^***	***P. multocida* CCUG 17976*^b^* × PM22 transconjugant*^c^***
**Tetracyclines**				
Oxytetracycline	0.250	16	0.125	16
Chlortetracycline	0.125	2	0.125	1
**Macrolides**				
Tylosin	32	256	32	256
Tilmicosin	4	64	2	64
Gamithromycin	2	64	1	64
Aminoglycosides				
Spectinomycin	64	1024	64	1024
Neomycin	16	256	4	256
**β-lactams**				
Penicillin G	0.125	0.125	0.125	0.125
Ampicillin	0.250	0.250	0.125	0.125
Ceftiofur	0.125	0.125	0.125	0.125
**Sulfonamides**				
Sulfamethazine	128	1024	128	1024
Sulfamethoxazole	128	512	128	1024
**Others**				
Enrofloxacin	0.250	0.250	0.250	0.250
Tiamulin	8	8	4	8
Clindamycin	2	1024	4	1024
Florfenicol	1	1	1	1

**^*a*^*Minimum inhibitory concentration (MIC). The highest MIC resulting from three experiments is given. *^*b*^*MIC for spontaneous rifampicin-resistant mutant (Mutant MIC was equivalent to WT for all antimicrobials tested). *^*c*^*Transconjugants were selected for with 10 mg/L of tetracycline.*

**TABLE 2:** Results of checkerboard synergy testing (FICIs) with non-susceptible antimicrobial combinations.

**Antimicrobial**	**Range*^a^* (mg/L)**	**MIC_ALONE_*^b^* (mg/L)**	**Combination FICI_MEAN_ ± SD (FICI_LOW_)*^c^***				
			
			**Spectinomycin**	**Tilmicosin**	**Tylosin**	**Sulfamethazine**	**Synergy (FICI_MEAN_ ≤0.5)**
***Mannheimia haemolytica* ATCC 33396 × PM22 transconjugant**							
Oxytetracycline	1–64	16	0.84 ± 0.23 (0.63)	0.57 ± 0.24 (0.31)	0.98 ± 0.22 (0.63)	0.99 ± 0.17 (0.75)	No interactions
Spectinomycin	64–4096	1024		0.51 ± 0.30 (0.25)	0.76 ± 0.63 (0.31)	0.74 ± 0.65 (0.28)	No interactions
Tilmicosin	4–256	32			0.60 ± 0.28 (0.38)	0.54 ± 0.09 (0.38)	No interactions
Tylosin	16–1024	256				0.63 ± 0.09 (0.53)	No interactions
Sulfamethazine	32–2048	1024					No interactions
***Pasteurella multocida* CCUG 17976 × PM22 transconjugant**							
Oxytetracycline	1–64	16	1.23 ± 0.18 (1.06)	0.58 ± 0.05 (0.50)	6.59 ± 5.82 (2.00)	1.21 ± 0.18 (1.03)	No interactions
Spectinomycin	64–4096	1024		0.60 ± 0.28 (0.38)	0.59 ± 0.28 (0.38)	1.11 ± 0.09 (1.00)	No interactions
Tilmicosin	4–256	32			0.58 ± 0.28 (0.38)	0.93 ± 0.25 (0.56)	No interactions
Tylosin	16–1024	256				0.84 ± 0.23 (0.56)	No interactions
Sulfamethazine	32–2048	1024					No interactions

**^*a*^*Range of antimicrobial concentrations tested. *^*b*^*Minimum inhibitory concentration (MIC) of antimicrobial tested alone. *^*c*^*Fractional inhibitory concentration index (FICI). The FICI values are given as the mean ± standard deviation resulting from three experiments. FICI values in brackets indicate the lowest value for the given combination. Synergism: ≤0.5; No interaction, ≥0.5.*

**TABLE 3:** Results of checkerboard synergy testing (FICIs) with non-susceptible and susceptible antimicrobial combinations.

**Antimicrobial**	**Range*^a^* (mg/L)**	**MIC_ALONE_*^b^* (mg/L)**	**Combination FICI_MEAN_ ± SD (FICI_LOW_)*^c^***			
			
			**Oxytetracycline**	**Spectinomycin**	**Tylosin**	**Synergy (FICI_MEAN_ ≤0.5)**
***Mannheimia haemolytica* ATCC 33396 × PM22 transconjugant**						
Penicillin G	0.0078–0.5	0.125	1.11 ± 0.09 (1.00)	0.59 ± 0.25 (0.25)	0.65 ± 0.09 (0.56)	No interactions
Enrofloxacin	0.0078–0.5	0.250	0.64 ± 0.30 (0.31)	0.70 ± 0.29 (0.38)	0.57 ± 0.23 (0.31)	No interactions
Florfenicol	0.0625–4	1	0.68 ± 0.22 (0.50)	0.61 ± 0.24 (0.38)	0.81 ± 0.23 (0.56)	No interactions
Ceftiofur	0.0313–2	0.125	1.28 ± 0.18 (1.06)	1.28 ± 0.18 (1.06)	3.29 ± 1.23 (1.50)	No interactions
Chlortetracycline	0.0625–4	1	1.21 ± 0.18 (1.03)	1.05 ± 0.51 (0.56)	0.57 ± 0.24 (0.31)	No interactions
***Pasteurella multocida* CCUG 17976 × PM22 transconjugant**						
Penicillin G	0.0078–0.5	0.125	0.93 ± 0.25 (0.56)	0.65 ± 0.09 (0.56)	1.13 ± 0.10 (1.00)	No interactions
Enrofloxacin	0.0078–0.5	0.250	1.23 ± 0.18 (1.06)	0.98 ± 0.22 (0.63)	0.99 ± 0.20 (0.63)	No interactions
Florfenicol	0.0625–4	1	0.74 ± 0.25 (0.50)	0.53 ± 0.09 (0.56)	0.61 ± 0.24 (0.38)	No interactions
Ceftiofur	0.0313–2	0.125	1.14 ± 0.11 (1.00)	1.28 ± 0.18 (1.06)	1.14 ± 0.11 (1.00)	No interactions
Chlortetracycline	0.0625–4	1	1.13 ± 0.10 (1.00)	1.13 ± 0.10 (1.00)	0.61 ± 0.24 (0.38)	No interactions

**^*a*^*Range of antimicrobial concentrations tested. *^*b*^*Minimum inhibitory concentration (MIC) of antimicrobial tested alone. *^*c*^*Fractional inhibitory concentration index (FICI). The FICI values are given as the mean ± standard deviation resulting from three experiments. FICI values in brackets indicate the lowest value for the given combination. Synergism, ≤0.5; No interaction, ≥0.5.*

## Discussion

Many ICEs site-specifically insert into tRNAs and, in general, are not able to change their location within the same host cell (Burrus et al., 2002; Michael et al., 2011b). Previous research has proven that ICE*Pmu1* and other ICE*Mh1*-like elements integrate into identical or near identical tRNA-leu (Michael et al., 2011b; Eidam et al., 2014; Beker et al., 2018; Bhatt et al., 2018). Here, we confirmed these findings, and provided further explanation for the tRNA-leu^UUG^ integration specificity of ICE*Mh1*-like elements. The ICE*Mh1*-like direct repeat is highly conserved because it is the basis for the tRNA-leu^UUG^ anticodon loop. Likewise, the *attL* site is conserved because it forms the tRNA’s TψC loop. Unexpectedly, the *attR* site does not conserve the tRNA’s D arm and loop. We speculate that this is the reason that integration seems to result in one functional and one disrupted tRNA-leu^UUG^ as the terminus with *attL* and the *attL*-associated direct repeat maintains the complete tRNA, but the *attR* terminus does not complete the second tRNA fragment. Examination of several genomes with ICE*Mh1*-like elements suggests this is the most frequent outcome of ICE insertion, but there are also examples where the ICE is flanked by complete tRNA-leu^UUG^, as well as ICE insertions where a terminus (i.e., the direct repeat) cannot be identified. We speculate that these represent on-going recombination post-integration, and may be mechanisms through which ICEs become non-mobile, or acquire new genes from the host chromosome.

The highly conserved structural features of tRNAs evidently provide an excellent site for exploitation by ICEs and other mobile genetic elements. Many ICEs from different bacterial species integrate into various tRNAs (Burrus et al., 2002; Johnson and Grossman, 2015; Castillo et al., 2017). There are several possible advantages to this. Firstly, all known life uses tRNAs, and organisms encode multiple tRNAs for specific amino acids (i.e., the basis of redundancy in the genetic code). Although specific tRNAs may be absent or altered, they are still widely present in multiple bacterial species and provide for a broad host range. Secondly, tRNAs have redundancy, so ICE insertion into a tRNA need not be lethal even if the tRNA is disrupted. This would facilitate the transmission of the mobile genetic element without exerting a deleterious effect on the host, which might be expected if the insertion site was a highly conserved and a non-redundant housekeeping gene. However, although single tRNA deletions may have no appreciable phenotypic effects under certain conditions, some tRNAs for a particular amino acid are known to contribute differently to cellular fitness in challenging conditions (Bloom-Ackermann et al., 2014). Thirdly, the structural features of tRNAs provide for conserved recombination sites. Although relatively short sequences (<90 bp), tRNAs contain multiple stretches of complementary base pairs that provide tertiary structure, enabling interactions with amino acids, mRNA, and the ribosome. In the case of ICE*Mh1*-like elements and tRNA-leu^UUG^, the 11 bp that constitute the ICE’s direct repeat and the tRNA’s anticodon loop were conserved with 100% identity in all ICE*Mh1*-like-containing genomes. Exploiting the anticodon loop in this manner also likely enables a broader host range compared to more variable features of the tRNA. Likewise, in the case of tRNA-leu^UUG^, we speculate that other conserved bp in the tRNA provide for the *attL* and *attR* recognition and/or recombination sites of the putative XerCD tyrosine recombinases. Although we did not explore that possibility here, this would be consistent with known XerCD mechanisms (Castillo et al., 2017).

It is difficult to accurately assess the potential host range of ICE*Mh1*-like elements. Here, we identified bacterial genera potentially receptive to the ICE based solely on the presence tRNA-leu, further refined by eliminating hits that did not contain the exact direct repeat and palindrome conserved at the *attL* terminus. As expected, related Pasteurellaceae and γ-Proteobacteria were most frequently represented, but genera from all Proteobacteria classes contained a receptive tRNA-leu. Most Gram-positive organisms may not be receptive to ICE*Mh1*-like elements, but some Bacilli and Clostridia did harbor a receptive tRNA-leu. We attempted to transfer ICE*Mh1*^PM22^ to *Clostridium difficile*, but did not successfully recover transconjugants (not shown). The host range of other conjugative elements in nature has been shown to be narrower than that observed in *in vitro* experiments (Rice, 1998). There are also other factors that could prevent ICE*Mh1*-like element transmission to bacteria, despite the presence of a receptive tRNA-leu. These may include phage (Lin et al., 2011), certain metabolites (Cabezón et al., 2017), and CRISPR-Cas systems (Zhang et al., 2013). Furthermore, ICEs transfer to recipients relatively infrequently, remaining silent and integrated, with excision and horizontal transmission occurring at low frequencies within the population (Delavat et al., 2017). The putative host range of ICE*Mh1*-like elements identified here includes many pathogens capable of causing significant human and veterinary disease. The transfer of AMR determinants into such bacteria could significantly compromise antimicrobial therapy.

Despite a fitness cost in the absence of antimicrobial selection, we also found that ICE*Mh1*^PM22^ transconjugants do not lose the ICE after long-term passage. This is likely due to ‘addiction’ *via* the action of toxin-antitoxin genes common to most ICE (Harms et al., 2018). For practical purposes this implies that the acquisition of an AMR-carrying ICE is not reversible at the single-cell level. Given that ICE*Mh1*-like elements may have a fitness cost to the host cell in the absence of antimicrobials, elimination of antimicrobial usage could help non-carriers to outcompete hosts. However, ICE*Mh1*-like elements frequently contain metal-resistance and other genes conferring benefits under dynamic selective pressures *in vivo*, so elimination of antimicrobial pressure may not be effective at dampening ICE-carrying populations. We also used ICE*Mh1*^PM22^ transconjugants to test for synergistic combinations of antimicrobials, hoping to identify combinations that might be effective against AMR BRD pathogens, potentially reducing antimicrobial usage and restoring drug effectiveness. Ultimately, we did not detect any synergistic interactions between the drugs tested using these organisms. This was not unexpected, and illustrates the relatively limited therapeutic options available to veterinarians for BRD treatment. The presence of multiple AMR determinants conferring resistance to most drug classes in these elements also underscores the dwindling effectiveness of antimicrobials. In conclusion, our results suggest that there is no easy solution to the emerging problem of AMR ICE in BRD pathogens using current antimicrobials and usage practices.

## Data Availability Statement

The PM22 sequence was deposited in GenBank under accession number CP045724.

## Author Contributions

AC and TM: conceptualization. AC and RZ: methodology, software, validation, formal analysis, and data curation. AC: investigation, visualization, writing, and original draft preparation. TM: resources, supervision, project administration, and funding acquisition. AC, RZ, and TM: writing, review, and editing.

## Conflict of Interest

The authors declare that the research was conducted in the absence of any commercial or financial relationships that could be construed as a potential conflict of interest.
